# GRACE: no place for doubts? An endorsement for communicating uncertainty in scientific assessments

**DOI:** 10.1007/s00204-016-1841-5

**Published:** 2016-10-11

**Authors:** Markus Woegerbauer, Walter Stepanek, Werner Brueller

**Affiliations:** Department for Integrative Risk Assessment, Data and Statistics, Austrian Agency for Health and Food Safety, Spargelfeldstrasse 191, 1220 Vienna, Austria

Over the past few years, Archives of Toxicology has provided excellent editorial coverage of the GRACE project and has issued—in accordance with the coordinators of the project—an invitation to an open scientific discussion on project-related subjects at several occasions (Hengstler 2015).

We are now taking the opportunity to enter the forum by providing a comment in response to the final “Conclusions and Recommendations” published online by the GRACE consortium (GRACE Consortium 2015). The amount of data collected by the project team and the efforts proficiently undertaken to solve the posed research questions are impressive and undisputedly acknowledged. However, it is astonishing that in this final report, the GRACE consortium appears to have refrained from communicating uncertainties which intrinsically affect any scientific analysis and subsequently the quality and validity of the drawn conclusions.

This observation is of special relevance when considering the envisioned primary target group of these final “Conclusions and Recommendations”: In contrast to peer-reviewed publications of the GRACE consortium and as vaguely connoted in the heading of their booklet, this contribution appears to be primarily tailored to address risk managers, policy makers, and decision makers.

However, this group of stakeholders is essentially relying on a comprehensive uncertainty analysis accompanying disseminated conclusions and recommendations for an informed decision making. Only if this provision is met, risk managers, policy makers, and decision makers are able to fulfill their tasks and duties responsibly. Uncertainty comprises all types of limitations in the knowledge available to assessors at the time an assessment is conducted and within the time and resources available for the assessment (EFSA Scientific Committee 2016). EFSA recommends that “*assessments must say clearly and unambiguously what uncertainties have been identified and what is their impact on the overall assessment outcome*.” This information is missing in the respective booklet. EFSA has recognized uncertainty analysis as a core element of transparency for all assessments and is of the opinion that “*…as a general principle assessors are responsible for characterising uncertainty, while decision*-*makers are responsible for resolving the impact of uncertainty on decisions. Resolving the impact on decisions means deciding whether and in what way decision*-*making should take account of the uncertainty. Therefore, assessors need to inform decision*-*makers about scientific uncertainty when providing their advice*” (EFSA Scientific Committee 2016). We are in line with EFSA in this respect. In the present case, the GRACE consortium and/or its representatives take on the role as assessors and therefore are appealed for addressing uncertainties which inherently affect their analyses in their final “Conclusions and Recommendations,” in the same booklet. This would substantially increase also the validity of and the confidence in the proposed “recommendations.”

Additionally, we miss a clearly structured description of the “strengths and limitations” of 90-day feeding trials in rodents and alternative studies as announced in the introductory section of the final “Conclusions and Recommendations” (GRACE Consortium 2015). A structured compilation of benefits and drawbacks of these research tools would have been a valuable asset in relieving the tasks of risk managers, policy makers, and decision makers.

However, we are confident that the GRACE consortium will be able to deliver this missing—but vital—information on uncertainties inherent to their conclusions to the decision makers at the European Commission which has the obligation to review in 2016 particular provisions in the Commission Implementing Regulation (EU) No 503/2013, based upon the results of the GRACE project (European Commission 2013).

We also would like to take the opportunity to elucidate some of our concerns as outlined in an internal communication between competent authorities and the European Commission and published by the GRACE consortium online (GRACE Consortium 2016). GRACE has been paradigmatically constructive by involving stakeholders without reservations during most stages of the project. The transparent interaction and the open-minded dialogue between all involved parties during these phases of the project is appreciated indeed and acknowledged.

However, coming to the key presentation (“Conclusions and Recommendations”) of their project, GRACE appeared to have stopped halfway: Although stakeholder involvement is emphasized on several occasions in the booklet, GRACE refrains from reporting any discrepancies which had arisen during extended oral and written discussions on the project design and on the interpretation of the results. Decision makers who have not closely monitored the evolution of the GRACE project and whose principal source of information on the topic might only be this respective booklet may get the impression that there have not been any discordances. It is clear that the booklet is not the appropriate forum for an extended presentation of stakeholder concerns. But avoiding any indication of aberrant opinions introduces a certain but unnecessary bias to this presentation. Referring only to web pages, incomplete databases, stakeholder reports, and papers which in several cases have not yet been published—without indicating any discordance—provides in our opinion only an insufficient basis for informed decision making. We are convinced that it was not the intention of the GRACE consortium to create this impression deliberately, and are sure that they are willing to provide the necessary clarification for the risk management.

Additionally, concerning the relevance of 90-day whole food/feed studies in rodents, we are of the opinion that the final “Conclusions and Recommendations” suffer also from other shortcomings:

1. Limitations and weaknesses of the overall study approach (e.g., testing of only a single active principle; no empirical analysis of stacked events or GMOs with complex alterations of metabolic pathways or events coding for different modes of action) are not clearly *communicated*. However, this information is of crucial importance for risk managers and for an informed decision making (EFSA Scientific Committee 2016). GRACE is only referring once to “*intrinsic limitations*” inherent to 90-day whole food/feed studies in rodents without even explicitly elaborating on them in the booklet (GRACE Consortium 2015).
2. The “Conclusions and Recommendations” imply general validity for the toxicological assessment of plant-derived GMOs, but the project design was not intended to provide experimental evidence in support of these generalizations. “*GRACE is expected to provide sound conclusions and recommendations on the adequacy of the approaches tested* *in the frame* *of GRACE*” (see p. 20; (GRACE Consortium 2015)) and, thus, is in the position to report results and conclusions on experience gained with MON810. But GRACE extrapolates far beyond this scope. This constitutes a bias which should be clearly *communicated* to the risk managers in the respective booklet as this is the basis for informed decision making.
3. GRACE highlights the importance of a “*targeted and testable hypothesis*” as trigger for animal experiments but does not *communicate* that unintended effects may be—in certain cases—unpredictable and unexpected and, thus, might not be detectable by a hypothesis-driven approach.
4. In our opinion, the selection of certain project partners was not as optimal as would have been required by the demanding specific conditions related to toxicity testing of GMOs in whole food and feed. In particular, the animal experimentation indicates some flaws which might have been relevant for the explanatory power of the results as a whole: The observation of circadian effects during trial A and trial B [compare p. 24 (GRACE Consortium 2015)] might be indicative for a non-optimal execution of these experiments, as this kind of effect would not have emerged if the studies were performed exactly according to OECD TG 408, EFSA 2011, and GLP requirements (EFSA 2011; OECD 1998). The necessity to include conventional control maize varieties (2 of them contaminated with MON810) as surrogates for missing historical control data is another indication for a non-optimal expertise on this type of whole food/feed studies in rodents.
5. A trend analysis considering trends and patterns in statistically nonsignificant differences (taking into account all applied diets, but not disregarding the primary importance of the comparison of GM and non-GM near-isogenic control groups) is missing, although this approach would provide valuable information. The analysis of trends is referred to in relevant guidelines (EFSA 2008).
6. In support of its conclusions, GRACE refers on several occasions to the CADIMA database and to (envisioned) scientific publications (GRACE Consortium 2015). At the time of writing, the CADIMA database was not fully functional and contained only the data from trial A and trial B (database last accessed: June 3, 2016). However, it is questionable whether a database lacking a substantial part of the project results and unpublished papers are the appropriate basis for risk managers to decide in a timely manner on possible amendments of Commission Implementing Regulation 503/2013 (European Commission 2013).
7. GRACE applied two conventional control maize varieties which were contaminated with MON810 in fact constituting GM diets with a GM content of approximately 1 % (Zeljenkova et al. 2014). According to the European GMO legislation currently in force, these diets would have had to be labeled as being genetically modified (European Commission 2003). So it is at least questionable whether these contaminated varieties are eligible to establish historical control data which should be mandatorily generated by non-GM lines (EFSA 2011).

The detailed line of argumentation related to our concerns as addressed above is provided as annex in the electronic supplemental material.

We do believe that the GRACE project provides a valuable contribution in clarifying the relevance of 90-day whole food/feed studies in rodents for the risk assessment of transgenic plants intended for commercialization and that GRACE stands exemplary for stakeholder involvement in scientific research of immanent public interest.

But we are also of the opinion that the obtained results and the derived conclusions should be communicated in a way that also uncertainties, shortcomings, drawbacks, and limitations of the overall experimental setup are clearly transparent and readily accessible for those in charge of public health: the policy makers and decision makers and last but not least the risk managers of national and international bodies.

## Electronic supplementary material

Below is the link to the electronic supplementary material.

Supplementary material 1 (PDF 260 kb)

## References

[CR2] EFSA (2008). Safety and nutritional assessment of GM plants and derived food and feed: the role of animal feeding trials. Food Chem Toxicol.

[CR3] EFSA (2011). Guidance for risk assessment of food and feed from genetically modified plants. EFSA Panel on Genetically Modified Organisms (GMO). EFSA J.

[CR4] EFSA Scientific Committee (2016) Guidance on uncertainty in EFSA scientific assessment. Draft. Available from https://www.efsa.europa.eu/sites/default/files/consultation/150618.pdf

[CR1] European Commission (2003). Regulation (EC) 1830/2003 of the European Parliament and of the Council of 22 September 2003 concerning the traceability and labelling of genetically modified organisms and the traceability of food and feed products produced from genetically modified organisms and amending Directive 2001/18/EC. Off J Eur Communities.

[CR5] European Commission (2013) Commission Implementing Regulation (EU) No 503/2013 of 3 April 2013 on applications for authorisation of genetically modified food and feed in accordance with Regulation (EC) No 1829/2003 of the European Parliament and of the Council and amending Commission Regulations (EC) No 641/2004 and (EC) No 1981/2006 Text with EEA relevance. Available from http://eur-lex.europa.eu/legal-content/EN/TXT/?uri=CELEX:32013R0503

[CR6] GRACE Consortium (2015) Conclusions and recommendations on animal feeding trials and alternative approaches and on the use of systematic reviews and evidence maps for GMO impact assessment. http://www.grace-fp7.eu/de/content/grace-published-its-final-conclusions-and-recommendations

[CR7] GRACE Consortium (2016). Manuscript available from http://www.grace-fp7.eu/de/content/open-letters-response-criticism-raised-austrian-authorities

[CR8] Hengstler JG (2015). Invitation to an open scientific discussion. Arch Toxicol.

[CR9] OECD (1998) Test No. 408: repeated dose 90-day oral toxicity study in rodents. OECD Publishing, Paris. Available from http://www.oecd-ilibrary.org/environment/test-no-408-repeated-dose-90-day-oral-toxicity-study-in-rodents_9789264070707-en

[CR10] Zeljenkova D, Ambrusova K, Bartusova M (2014). Ninety-day oral toxicity studies on two genetically modified maize MON810 varieties in Wistar Han RCC rats (EU 7th Framework Programme project GRACE). Arch Toxicol.

