# Supplementary material for: GRACE: no place for doubts? An endorsement for communicating uncertainty in scientific assessments
Source: Arch Toxicol. 2016 Oct 11;90(12):3139–41. doi: 10.1007/s00204-016-1841-5 (PMC5104811; doi:10.1007/s00204-016-1841-5)
Supplement: Supplementary file 1 — Supplementary material 1 (PDF 260 kb) [file 204_2016_1841_MOESM1_ESM.pdf]

Statement on "Conclusions and Recommendations: Part I" of the GRACE Consortium and Reply to the GRACE Response on our Contribution from February 2016

GRACE provides a valuable contribution and an impressive amount of hard facts eagerly awaited in the dispute about the relevance of 90-day whole food/feed studies for GMO risk assessment. The efforts to generate empirical data to close knowledge gaps and fuel an emotional discussion of immanent public interest with insights from laboratory experimentation are remarkable and certainly worthwhile. GRACE has been constructive by involving stakeholders in a research project of public interest and has been exemplary for a transparent interaction and an open-minded dialogue between the involved parties. This all is appreciated indeed.

However, coming to the core presentation of their project ("Conclusions and Recommendations" (1)) GRACE appeared to have stopped halfway on their mission to ensure transparency: Although stakeholder involvement is emphasized on several occasions in the booklet, GRACE shows no intention to report uncertainties inherent to the project design and discrepancies which had arisen during extended oral and written discussions on the project execution and interpretation of the results. This observation is of certain relevance when considering the envisioned primary target group of these final "Conclusions and Recommendations": risk managers, policy- and decision makers. Especially this group of stakeholders is essentially relying on a comprehensive uncertainty analysis accompanying disseminated conclusions and recommendations for an informed decision-making (2). EFSA regards information on uncertainty inherently accompanying results, conclusions and recommendations of any scientific assessment as a core element of transparency for all assessments and is of the opinion that assessors need to inform decision makers about scientific uncertainties when they provide their advice (i.e. their "recommendations") (2). We are here completely in line with EFSA.

Decision makers who have not closely monitored the evolution of the GRACE project and whose principal source of information on the topic might only be this respective booklet may get the impression that there have not been any discordances on the project design and the interpretation of the results between project management and some stakeholders. It is clear that this booklet is not the appropriate platform for an in-depth discussion of stakeholder concerns. But the absence of any indication of concerns expressed during extensive written and oral consultations on the project and its outcome creates an unnecessary bias. This is not an optimal basis for an informed decision making. Nevertheless we are confident that GRACE will communicate the missing clarifications for an informed decision making to the risk managers in charge for this issue at the European Commission.

GMO risk assessment has been now in the focus of public interest for already several decades. Considering the potential magnitude of exposure of humans, animals and the environment (i.e. worldwide, lifelong) the request for a thorough safety testing of GMOs and derived products before commercialization is justified from a consumer perspective and undisputed current consensus in the GMO legislative framework of the European Union (3, 4). This approach guarantees a high level of product safety and should provide the basis for consumer confidence into products derived from the food/feed chain. The tools and procedures to achieve this goal are also laid down in several guidance documents prepared by EFSA which foresee under certain circumstances the implementation of animal tests for obtaining a comprehensive toxicological profile of the GMO or the diet containing or consisting of that GMO (5-7). Due to current technical limitations it is doubtful whether at present in vitro test systems can provide equivalent information in relation to animal experiments (8, 9). On the other hand the mandatory performance of 90-day whole food/feed studies in rodents and the significance of the results obtained by an untargeted approach are also under debate (10). This stalemate is far from being satisfactory. However, whatever kind of risk assessment tool for the evaluation of GMOs is applied one consideration has paramount: the guiding principle of product and consumer safety must not be compromised at any rate.

To obtain meaningful and to avoid misleading false negative results from 90-day whole food/feed studies laboratory-induced variation - which may mask potential adverse effects - has to be kept to its absolute possible minimum. Hence, we are of the opinion that the experimentations ideally should be performed by a highly skilled team of laboratory workers and experts with a longstanding expertise and proficiency in the execution of 90-day whole food/feed studies in rodents.

Concerning the final report of the GRACE consortium ("Conclusions and Recommendations" (1)) and the accompanying publication of Zeljenkova et al. (i.e. trial A and trial B of the 90-day whole food/feed studies in rodents) (11) we have identified the following inadequacies which we believe are warranted to be communicated by the GRACE consortium in support of risk managers and policy makers to facilitate informed decision making on the issue:

1. The report is lacking an **uncertainty** analysis - although information about uncertainty in their analyses is a key task for assessors when giving their advice (2).
2. GRACE appears to refrain from **communicating** limitations and shortcomings of their overall study approach in their final "Conclusions and Recommendations" – a crucial information for risk managers but missing for an informed decision making (2).
3. The "Conclusions and Recommendations" imply general validity for the toxicological assessment of plant-derived GMOs, although GRACE has obtained empirical data exclusively for MON810. Extrapolations from this starting point should be clearly indicated and **communicated** transparently to the risk managers in this booklet.
4. The selection of certain project partners appears to have not been as optimal as being adequate for the demanding conditions relating to toxicity testing of GMOs with whole food and feed. Particularly concerning the animal experimentation there have been some weaknesses which might have been relevant for the interpretation of the results as a whole: The observation of circadian effects during trial A and trial B (compare p. 24 (1)) might be indicative for a non-optimal execution of these experiments, as this kind of effect would not have emerged if the studies were performed exactly according to OECD TG 408, EFSA 2011, and GLP requirements (6, 12, 13). The necessity to include conventional control maize varieties (2 of them contaminated with MON810) as surrogates for missing historical control data is another indication for a non-optimal expertise on this type of whole food/feed studies in rodents.
5. A trend analysis considering trends and patterns in statistically non-significant differences (taking into account all applied diets but not disregarding the core comparison between verum and near-isogenic control group) is missing although this approach would provide valuable information.
6. In support of its line of argumentation GRACE refers on several occasions to the CADIMA database and to envisioned scientific publications (1). At the time of writing the CADIMA database was not fully functional and contained only the data from trial A and B (database last accessed: June 3rd, 2016). However, it is questionable whether a database lacking a substantial part of the project results and unpublished papers can be an appropriate basis for risk managers to decide in a timely manner on possible amendments of Commission Implementing Regulation 503/2013 (14).
7. Two conventional control maize varieties were contaminated with MON810 in fact constituting a GM diet containing approx. 1% GM (please see table 2 of (11)). According to the European GMO legislation currently in force these diets would have to be labelled as being genetically modified (15). So it is at least questionable whether these contaminated varieties are eligible to establish historical control data which should be generated mandatorily by non-GM lines (6).

A detailed line of argumentation in support of our concerns can be found in the attached Annex.

ANNEX

Please find below our feedback and clarifications to the GRACE commentary on some of our concerns raised in our initial contribution in response to the final report of the GRACE consortium ("Conclusions and Recommendations" (1)). The initial correspondence can be found on the GRACE homepage (http://www.grace-fp7.eu/en/content/).

1. Reply to the GRACE response to our comment 1 on the relevance of historical control data and the role of conventional varieties for the evaluation of 90-day feeding studies.

The intention of our initial comment worded in general terms was to indicate that the comparison between verum and control (in this case the non-GM near isogenic control) is the core element of the evaluation of *in vivo* toxicological tests, among them 90-day feeding studies. If statistically significant differences occur during this comparative process they have to be considered in depth and with professional care (16).

Our evaluation was focused on trial A and B data where many differences in different parameter groups (e.g. body weights, body weight gains; statistically significant: clinical pathology, organ weights) became apparent (11). Remarkably, all of these differences between GM and non-GM fed groups were considered toxicologically irrelevant. Many of those were neglected or dismissed by the GRACE consortium by arguing that the magnitude of the effect was still within "historical controls" as ultima ratio. Regarding the relative pancreas weights for instance in trial A (male verum groups with significantly lower values compared to control; control : 11% verum : 33% verum : conventional 1 : conventional 2 0.141 ± 0.016 : 0.115 ± 0.016 : 0.112 ± 0.009 : 0.134 ± 0.026 : 0.121 ± 0.022) the differences occurred not only against control but also against reference groups fed with conventional maize varieties under identical test conditions (11).

And even if these deviations might actually not imply pathological processes or are in fact not attributable to the test material they may be indicative for a sub-optimal execution of the study: Considering the generally very low concentrations of the test material in the diets a reduction of laboratory-induced variation to the absolute possible minimum is mandatory. The small applied dosages probably induce only minute effects which may be masked by background incidence. It is also evident that a higher number of varieties included into an experiment increases variability and reduces the likelihood of statistically significant differences.

The GRACE consortium insinuates in an additional statement (see comment 15.9) that we overestimate the role of conventional controls for the evaluation of toxicologically relevant results under certain circumstances. We would like to emphasize again that in toxicological risk assessments – also with GMOs - predominantly the dose groups have to be compared with the control which is kept under the same conditions but without the interesting/test substance/s. But if – for whatever reasons – some other groups are employed, we are of the opinion that it is justified to take a look at these data and check them also for potentially emerging trends (e.g. ranging over all other groups as against the verum groups or vice versa and, thus, distinguishing the test groups from all other groups). The GRACE consortium itself takes advantage of conventional control data to dismiss – inter alia – statistically significant differences between test groups and the non-GM near isogenic control as not pathologically relevant so these data should be also eligible for the evaluation of (also

statistically non-significant) tendencies in the relevant data packages. This approach would provide valuable additional insights and should be systematically considered for the sake of gaining as much information as possible from the experiment.

2. Reply to the GRACE response to our comment 2 on the dismissal of statistically significant differences supported by trends appearing also in conventionally fed controls, the dismissal of gender-specific effects and the availability of study results from trial C.

Referring to the Disclaimer on the cover page of the supplement made available to a certain audience and containing information on trial C stating that the one-year oral toxicity study on a genetically modified maize MON810 variety in Wistar Han RCC rats (= trial C) "*contains unpublished data, interpretations, conclusions and recommendations* ***all of which*** *are* ***preliminary*** *and might be* ***subjected to changes"*** and that "*due to the* ***preliminary nature*** *the authors reserve the right* ***not*** *to be* ***responsible*** *for* ***correctness and completeness*** *of the information provided"* (17) and considering the general Non-Disclosure Agreement which had to be signed for granting access to the data sets emphasizing that "*The Confidential Information subject to this Agreement is made available „as such" and* ***no warranties*** *of any kind are granted or implied with respect to the* ***quality*** *of such information including but not limited to, its applicability for any purpose, non-infringement of third party rights,* ***accuracy, completeness or correctness****"* (18) trial C data were not evaluated.

It is not our obligation to analyze and interpret data which are "**preliminary**" and "**subject to changes**" and for which the authors grant **no guarantee** concerning their "**quality**", "**accuracy**", "**completeness**" or "**correctness**" for official communications between competent authorities and notified risk assessment bodies. At the time of presentation the raw data sets were not complete, a deficiency which was conceded by the GRACE team only after intense discussions during the respective conference in Vienna (October 2015). Consequently, trial C data were not evaluated.

In contrast to trial C, trial A and trial B data have been published in a peer-reviewed journal, are subject to unrestricted public scientific scrutiny, and therefore have been evaluated by our team.

The GRACE team argues in its response to our request to pay attention to gender-specific results that effects could not be detected in both sexes, e.g. increased blood glucose appeared only in male rats of trial A, not in females. Moreover, sex-dependent effects (e.g. neutrophils, thymus weight) are subliminally downgraded as being not applicable to the whole species. But it is a well-known fact that males and females may react physiologically (and pathologically) different. Clayton and Collins indicate that "*…Publications often continue to neglect sex-based considerations and analyses in preclinical studies. Reviewers, for the most part, are not attuned to this failure. The over-reliance on male animals and cells in preclinical research obscures key sex differences that could guide clinical studies. And it might be harmful: women experience higher rates of adverse drug reactions than men do. Furthermore, inadequate inclusion of female cells and animals in experiments and inadequate analysis of data by sex may well contribute to the troubling rise of irreproducibility in preclinical biomedical research…" (19).*

Therefore, attention has to be paid even when an effect is only seen in one gender.

As already stated, serum glucose levels were elevated with both *verum* groups of male rats, and this not only against control but also against both conventional groups. The observation correlated well with decreased pancreas weights of these *verum* groups. This could indicate some insulin deficiency (20). And taking further into consideration that a considerable body of evidence indicates that the release of glucose by the liver and kidney are interrelated so that a reduction in release by one organ is associated by an increase by the other to further maintain optimal glucose homeostasis (referred to as hepatorenal glucose reciprocity) (21, 22), and that insulin deficiency leads to stimulation of renal gluconeogenesis (23), some suspicious facts cannot be excluded that substance effects have been disbalancing liver-pancreas-kidney axes. These suspicions cannot be absolutely eliminated by liver (e.g. aminotransferases) and kidney (e.g. creatinine) data which were roughly within normal ranges or showed values which were not correlated to adverse effects (creatinine), at least at first glance. It is well known that in the first stages of disease development sensitive parameters may respond (long before gross or histo-pathological manifestations occur) which are compensated and perhaps overcompensated afterwards for a certain duration of the treatment (maybe until after the completion of a medium-term experiment). And glomerular filtration rate and insulin have not been measured.

With trial B, red blood cell counts are significantly elevated, both in male and female *verum* rats, in both cases the same trend can be seen against both conventional groups. The GRACE team argues in their response that such findings have not been seen in the other trials, including the 1-year feeding study. Generally speaking, apart from distinct effects (e.g., this observation originates not from biological variations but from substance-related reasons disturbing the homeostasis) which occurred just in trial B this incident is certainly not meant to demonstrate a *lege artis* performance of the trial. And with these low doses of the active principle(s) and relatively short exposure periods someone has always to bear in mind that even slight deviations may have a detrimental background.

Secondary acquired erythrocytosis which is normally erythropoietin-mediated can result from central or local hypoxia, renal or hepatic imbalances/diseases (24). The observed increases of red blood cell counts – even when not massive – may give indication of dysregulations at a subclinical stage.

Regarding the decrease in the percentage of neutrophils and the increase in the percentage of monocytes in female *verum* rats with trial B, the GRACE team argues with conflicting data in the individual trials, decreases or increases and no changes at all, but certainly without a general trend. But they pay no attention to the fact that decrease in the percentage of neutrophils and increase in the percentage of monocytes correlate well because neutropenic states are often accompanied by monocytosis (25) having a clinical significance. And should these effects be outliers and not indicating a general trend this would be, as already said, not a good demonstration of the reliability and reproducibility of the test conditions.

With regard to the increases in thymus weights of females in trial B the GRACE team argues again that these were isolated results and not seen in other trials. But they ignore the indications for instance seen by Finamore et al. (26) with mice that MON810 maize induced alterations in the percentage of T and B cells and of CD4(+), CD8(+), gammadeltaT, and alphabetaT subpopulations of weaning and old mice fed for 30 or 90 days, respectively, at the gut and peripheral sites. And thymus is a specialized primary lymphoid organ of the immune system playing amongst others a decisive role in the further development of the thymocytes into T cells (27).

Other examples (not claiming any completeness) of the immunogenicity of MON810 are given by the following paper excerpts:

"*GM maize seemed to induce significant changes in white blood cell populations which are associated with an immune response*" (28).

"*We observed that IL-12 and IFNc production from mitogenic stimulated peripheral blood mononuclear cells decreased (P<0.10) following 31 days of GM maize exposure. While Cry1Ab specific IgG and IgA were not detected in the plasma of GM maize-fed pigs, the detection of the cry1Ab gene and protein was limited to the gastrointestinal digesta and was not found in the kidneys, liver, spleen, muscle, heart or blood… IL-6 and IL-4 production from isolated splenocytes were increased (P<0.05) in response to feeding GM maize while the proportion of CD4+ T cells in the spleen decreased. In the ileum, the proportion of B cells and macrophages decreased while the proportion of CD4+ T cells increased in GM maize-fed pigs. IL-8 and IL-4 production from isolated intraepithelial and lamina propria lymphocytes were also increased (P,0.05) in response to feeding GM maize….Alterations in immune responses were detected;…*"(29).

"*However, a residual effect on lymphocyte count was apparent in older pigs fed Bt maize in early life, an effect which was not present following long-term Bt or isogenic maize consumption.*"(30)

"*Exposure to purified Cry1Ab resulted in specific anti-Cry1Ab IgG1 and IgE production, indicating inherent immunogenicity and allergenicity. Mice exposed to leaf extracts from both MON810 and unmodified maize demonstrated influx of lymphocytes and eosinophils in the broncho-alveolar lavage, and increased cytokine release in mediastinal lymph node cells. The results indicate that the airway exposure to Cry1Ab proteins may be a route of practical relevance.*"(31)

3. Reply to the GRACE response to our comment 3 on extended blood sample testing and appropriate other test designs.

According to EFSA (32), 90-day studies in rodents should be conducted with the full range of observations as described in the OECD TG 408. Additional endpoints described in the more recent guideline on repeated-dose 28-day oral toxicity study in rodents (OECD TG 407) may be considered for assessment, depending on the nature of the food/feed being tested and the available information (33). Other endpoints should also be considered if there are indications that whole food/feed may have effects on e.g. the cardiovascular, nervous, gastrointestinal tract or immune system. For instance, if whole food/feed is expected to have an impact on the gut, then the microbial flora should be investigated. Additional markers of potentially adverse nutritional and/or metabolic effects should be considered on a case-by-case basis, according to the available body of evidence and the type of whole food/feed under investigation.

Thus, OECD TG 408 provides "*In addition, studies to investigate serum markers of general tissue damage should be considered*" (12).

Guzma´n-de la Garza FJ et al. (34) described as markers of non-specific cell damage aspartate aminotransferase, alanine aminotransaminase, and lactic dehydrogenase, as markers of inflammation tumor necrosis factor alpha, interleukin-6, and interleukin-1 beta, and as markers of

intestinal mucosal damage intestinal fatty acid binding protein and D-lactate. With the exception of the aminotransferases none of those parameters were measured.

OECD TG 407 expresses that "*In the international test program some evidence was obtained that subtle endocrine effects by chemicals with a low potency for affecting sex hormone homeostasis may be identified by disturbance of the synchronisation of the oestrus cycle in different tissues and not so much by frank histopathological alterations in female sex organs" (33).* Such measurements were not performed by the GRACE team.

The "Explanatory statement for the applicability of the Guidance of the EFSA Scientific Committee on conducting repeated-dose 90-day oral toxicity study in rodents on whole food/feed for GMO risk assessment" says that "*other haematological endpoints for consideration are the red blood cell mean cell volume (MCV), distribution width (RDW), reticulocytes absolute count. These are currently accepted standard parameters offering additional information on the haematological profile of the test system*" (35). Red Cell Distribution Width (RDW) and reticulocytes absolute count have not been analyzed by the experimenters.

Moreover, "*differential leukocyte count should be reported in absolute numbers and not as percentage of the total count.*" With the exception of lymphocytes this has not been done. And what is even more disadvantageous, even though the paper by Zeljenková et al. stated that "*for the differential leucocyte count, blood smears were stained with the May–Grunwald and Giemsa–Romanowski dyes and thereafter examined by light microscopy; the percentage of lymphocytes, neutrophils, eosinophils, basophils and monocytes were determined by examining 100 cells*" (11), no data on basophils were presented.

Like mast cells, basophils possess high-affinity immunoglobulin (Ig) E receptors (FceRI) that are cross-linked upon engagement of receptor-bound IgE with corresponding antigens ("allergens"), resulting in the release of a number of mediators that are in part common for both cell types. Nevertheless, findings have provided new insights into the possible role of basophils in allergic disease and immunity to pathogens. Most notably, the discovery that basophils rapidly produce large amounts of the regulatory cytokines interleukin (IL)-4 and IL-13, together with the constitutive expression of CD40L and CCR3 on their surface, has fueled speculations that extend beyond their recognized role as effector cells in IgE-mediated reactions (36).

Therefore, the lack of basophil data would be a substantial gap as - as already mentioned above - MON810 maize is suspected to mediate immunogenic effects (please see comment 2).

And last but not least, scientific assessments must include consideration of uncertainty (2). Assessments must say clearly and unambiguously what sources of uncertainty have been identified and what is their impact on the final assessment outcome: what range of outcomes is possible, and how probable they are (2). That prerequisite is completely ignored by the GRACE consortium.

4. Reply to the GRACE response to our comment 4 concerning triggering 90-day whole food/feed studies in the light of the RRR approach.

Regarding the original GRACE statement in section 5.1 of the "Conclusions and Recommendations" our concerns have to be rendered more precisely: The first bullet point of the GRACE argumentation

says "*Due to the intrinsic limitations of a feeding trial with whole food/feed, a mandatory performance cannot be justified in the light of the RRR approach based on the available science* (p. 31; (1))." The whole sentence is vague: GRACE does not specify what kind of "intrinsic" limitation the consortium is referring to, although this information is crucial for the risk manager for a well-informed decision-making (2). An explicit listing of these "intrinsic" limitations as well as a discussion of uncertainties of the overall approach is missing. Due to the only limited dosages of the GM-derived active principle/s in food and feed a 90-day study might show no measurable adverse effects. However, under bullet point 5.1 the GRACE consortium implies that this conclusion is generally valid, but does not provide empirical evidence in support for every type of feeding study or for any other possible constellation besides the tested MON810 varieties. We are concerned by the generalization the GRACE consortium is posting without providing relevant scientific evidence (i.e. derived from their experiments actually performed) in support of their assertions. We would like to remind that "*GRACE is expected to provide sound conclusions and recommendations on the adequacy of the approaches* ***tested in the frame*** *of GRACE*"(see p.20; (1)). Notably, the GRACE consortium refers to "*available science*". In the presented context this term is also ambiguous and misleading.

Additionally, we would like to draw the attention to the fact that for instance for food additives, which enter the human or animal body frequently and over long periods of time, a long-term exposure test is usually legally mandatory (37). There are also indications that minuscule amounts of certain agents which generate only slight or even no obvious acute effects may induce adverse effects only after constant long-term/life-long exposure. Thus, there may be a chance to neglect or underestimate the possibility of the occurrence of adverse effects by magnitudes not showing measurable effects after shorter exposure periods.

The third bullet point regarding section 5.1 says: "*Omics approaches on* ***plant*** *material may inform the development of a targeted hypothesis in the future in order to scientifically justify the performance of feeding trials with whole food/feed and to target the study design to the posed safety concern* (p. 31;(1))." Omics approaches on plant material may have indeed a promising potential for streamlining GMO risk assessment of whole food and feed in the future. However, at present these methodologies are **not** eligible for routine application in GMO risk assessment due to intrinsic limitations (e.g. uncertainties introduced by the selected statistical evaluation algorithms for profile generation, missing or incomplete databases for comparative assessment etc…) and a non-existing standardization of the testing regimens (9). This bullet point is therefore no valid line of argumentation in support of an omission of 90-day feeding trials in the light of the RRR approach in the present situation and no decision support for risk managers, who are requested to decide near-term (i.e. during 2016) on this topic (14). Market maturity and undisputed applicability of omics-technologies on plant materials in the GMO risk assessment area within this narrow time frame can be excluded. For a well-informed decision-making on this issue it is irrelevant whether the presented method may have some practical benefits in 5 or 10 years.

Ralf Einspanier & co-workers of GRACE Working Package 2 "in vitro studies-on animal samples" additionally indicate that "*Without any significant effects of GM-treatments in animal trials, a direct correlation between in vivo vs. in vitro systems is unfeasible" (38)*. This confirms the currently very limited utility of omics/in vitro studies in the area of GMO risk assessment also with animal tissues. A realistic extrapolation of the results obtained in these in vitro systems to the situation occurring in vivo (i.e. in the organism as a whole) appears to be impracticable at the present status of knowledge in the field, because of the only limited possibility to take into account the kinetics as absorption,

distribution, protein binding, metabolism, concentration at the target etc. Using omics and in vitro studies on i) plant material and/or ii) animal tissue as line of argumentation against animal feeding studies in the light of the RRR approach is premature and suffers presently from a poor scientific support.

The fourth bullet point regarding section 5.1 declares that *"The expected magnitude of a distinctly identified potential effect should be included into the test hypothesis and guide the decision whether a feeding study should/could be performed to achieve a valuable information gain"* (1). This euphonic but somewhat nebulous sentence may have two connotations: it may either i) imply that even if an effect is induced by the active principle this effect could be too small to be detected by the test system or ii) it might be not noteworthy at all. But as already indicated minuscule amounts of a substance may be able to induce adverse effects on human and animal health after long-term exposure.

GM products and tests with them, respectively, are premier examples for active principles presumably only applied in minute amounts (in the microgram range per animal). Under these conditions an utmost emphasis has to be placed on a well-experienced test strategy and performance keeping the non-substance-related variations at an absolute minimum. Compliance with the highest quality criteria have to be requested from the laboratory performing the necessary animal experimentation. Keeping laboratory-induced variations to their absolute minimum is a key imperative if meaningful results from 90-day feeding studies are to be expected (5). A core element for achieving this goal is the involvement of testing laboratories with a proven record for and a longstanding experience with 90-day feeding studies with GM plants in rats. The GRACE project shows some weaknesses in this respect: Historical control data for the necessary test system were not available (see Stakeholder Report Vienna, GRACE response No. 31 (38)). Substantial variations within some test parameters caused by circadian rhythm (see Stakeholder Report Vienna, GRACE response No. 62 (38)), which should not have occurred if the testing regimens were performed skillfully according to the established guidelines for 90-day feeding studies in rodents (because these guidelines are explicitly designed to exclude this kind of laboratory-induced variation (6, 12)), are prominent examples and an indication of inadequacies in the handling of the test animals and in coordinating the test procedures.

Moreover, in the fourth bullet point the GRACE consortium is dismissing again the phenomenon of unintended/unexpected effects which cannot be captured with the suggested "test hypothesis"-approach, because they are difficult to be foreseen by their nature (10).

5. Reply to the GRACE response to our comment 5 on the immature status of "-omics" approaches for GM risk assessment.

The GRACE consortium insinuates that we *"… tend to interpret the GRACE recommendations as a "new standard" in GM crop safety testing."* We would like to clarify that we are far off considering the GRACE recommendations as a new standard in GM crop safety testing and we welcome GRACE's intention to *"… promote a broader scientific discussion about potential test systems and their added value in the context of the GMO risk assessment."* starting with their evaluation of "omic"-technologies for applicability in this area.

But we would also like to indicate that even though it is not explicitly stated by GRACE "*that a targeted hypothesis tailoring the study design to a posed safety concern can only be developed by an omics approach*" (that is currently not validated) the GRACE consortium formulates under section 2.1 "*Thereby it (remark: GRACE omics data) provides a better basis for the decision on the scientific rationale to frame the subsequent risk assessment steps, which may include the performance of an animal feeding trial with the plant-derived whole food/feed* (p.27 (1))" which indeed strongly suggests a preference for applying "omics" techniques to decide if in vivo studies are indicated or not. This is premature as the correlation of the omics-derived results to effects on the whole living system is far from being conclusive at present (8). Some of the current limitations of "-omics" technologies (including their application on plant material and human/animal tissues, fluids, organs etc..) for risk assessment purposes amongst others are (8):

- For the identification of the mode of action, knowledge about the functions of affected genes is required. At present, however, the function is not yet known for a relatively large proportion of the genes affected by a treatment. These genes will be disregarded for biological interpretation while they will be included in the biomarker approach.
- In addition, many processes will have an overlap in genes. The biological interpretation of pathway analysis should thus be done with caution and be confirmed by biochemical or cytological experiments.
- Omics investigations that follow a traditional dose-response design appeared to be quite rare so that the translation of omics information into the regulatory risk assessment framework, or into other decision making, is particularly difficult.
- Most of the omics studies performed so far are not designed for the purpose of food safety risk assessment. So, a proper framework design for an omics study (e.g. the use of a range of doses to support dose-response modelling) is crucial to be able to perform (quantitative) risk assessment.
- Statistically significant correlations between omics and physiology have not yet been established.
- Most importantly, it must be noted that however promising all developments may be, they have not been realized in everyday food risk assessment (chemical or microbiological) to this date.

For additional clarifications please see our reply to comment 4.

6. Reply to the GRACE response to our comments 6-8 on shortcomings concerning statistical power, statistical models, animal test designs and alternative approaches pursued in the frame of GRACE.

We observed that the final "Conclusions and Recommendations" do not **communicate** potential limitations and weaknesses of the overall approach and the applied test designs (1). This is also relevant for the statistical methods used for analyzing the data from the GRACE animal feeding studies. It is insufficient to refer indifferently to stakeholder reports but omit to clearly address limitations, shortcomings and weaknesses in the final "Conclusions and Recommendations", in the same booklet. It is necessary to realize that "…*Understanding of the type and degree of uncertainties identified in the assessment helps to characterize the level of risk to the recipients and is therefore essential for informed decision-making*" (2). These are crucial points when considering the primarily addressed auditorium – risk managers and policy-makers who may not have followed the evolution

of the GRACE project in every detail but who have to interpret the "Conclusions and Recommendations" given by GRACE adequately.

Applying the most appropriate statistical methodology and the achieved power are important considerations in GM whole food/feed testing (39). Additionally, it has to be indicated that the discussion amongst the stakeholders regarding which statistical methodology was to be used in the GRACE studies was strongly diversified (see comments 19, 37, 68 of Stakeholder Report Brussels (40)). The statistical power with respect to animal feeding trials is mainly influenced by the size of the samples (number of animals), but also by reducing the measurement error by controlling of extraneous sources of variation and using more reliable measures of constructs (39). There are indications that the laboratory in charge of executing the animal experimentation appeared to have had problems keeping laboratory-induced variations to an absolute minimum (e.g. appearance of circadian effects documented in data packages which would not have occurred if the experiment had been performed according to the internationally agreed guidance (6, 12, 13); one-week breakdown of air condition in the animal housing (11); no experience with longer term whole food/feed studies with maize in rats which may have caused additionally logistic problems in handling higher numbers of animals simultaneously. Reducing laboratory-induced variation is of crucial importance due to the minute amounts of active principles and other potentially detrimental components in the test diets. Possibly induced effects by these small amounts may easily remain hidden and be masked by laboratory-induced variations.

7. Reply to the GRACE response to our comment 9 on the availability of trial C data.

The GRACE consortium asserts that concerning trial C "*…the complete data set (as means ± standard deviation of each endpoint) was available…"* to the Austrian colleagues and refers to a Non-Disclosure Agreement.

We would like to indicate that the Disclaimer on the cover page of the supplement which has been made available to a limited audience comprising information on trial C (= "1-year study") states that this one-year oral toxicity study on a genetically modified maize MON810 variety in Wistar Han RCC rats "*…contains unpublished data, interpretations, conclusions and recommendations* ***all of which*** *are* ***preliminary*** *and might be* ***subjected to changes***" and that "*due to the* ***preliminary nature*** *the authors reserve the right* ***not*** *to be* ***responsible*** *for* ***correctness and completeness*** *of the information provided"* (17). Moreover, the general Non-Disclosure Agreement which had to be signed for granting access to the data sets underlines that "*The Confidential Information subject to this Agreement is made available „as such" and* ***no warranties*** *of any kind are granted or implied with respect to the* ***quality*** *of such information including but not limited to, its applicability for any purpose, non-infringement of third party rights,* ***accuracy, completeness or correctness***" (18).

It is not our business to analyze and interpret data which are "**preliminary**" and "**subject to changes**", and for which the authors grant **no warrant** concerning their "**quality**", "**accuracy**", "**completeness**" or "**correctness**" in the light of official communications between competent authorities and notified risk assessment bodies, respectively. At the time of presentation the raw data sets were not complete, a deficiency which was conceded by the GRACE consortium only after intense discussions during the respective conference in Vienna (October 2015). Consequently, trial C data were not evaluated.

In contrast to trial C, trial A and trial B data have been published in a peer-reviewed journal, are subject to unrestricted public scientific scrutiny, and therefore have been evaluated by our team.

8. Reply to the GRACE response to our comments 10 - 12 concerning the applicability of non-targeted omics approaches (e.g. one–class model) for the risk assessment of GM crops.

We would like to add the following considerations to the explanations provided by the GRACE consortium in their response: Transcriptome analysis enables the determination of differences between plant varieties based upon the establishment of variety-specific transcription profiles. However, it is still open to scientific debate whether a possible difference in the plant variety specific transcription profiles is of toxicological relevance or not (41).

According to Van Dijk et al (41) the estimation whether a GM plant falls within the single class of "safe" plants is based on "*estimating the similarity of new varieties to a reference baseline class of known safe varieties"*. It is not clear whether transcription "profiles" are informative enough to facilitate the detection of small aberrations (quantitative as well as qualitative) responsible for unintended or even unexpected effects. To achieve this goal large databases for transcriptome data are a prerequisite but have not been comprehensively established, yet, to allow reliable comparative assessments.

9. Reply to the GRACE response to our comment 13 concerning the absence of a description of the limitations, shortcomings, weaknesses and uncertainties inherently affecting risk assessments in the final "Conclusions and Recommendations".

We have to reiterate the fact that the grade of uncertainty in the line of reasoning by the GRACE consortium is not reported in the final "Conclusions and Recommendations" and limitations in the overall test approach which may have severe impact on the validity of the presented conclusions are not addressed (1). However, these are crucial requirements for an informed decision-making (2). Considering the target of these "Conclusions and Recommendations" – risk managers and policy-makers who are usually not scientific experts in the field – GRACE has the obligation to communicate the grade of uncertainty of their analyses and to discuss limitations and shortcomings inherent to the applied test approaches.

Other drawbacks are at first sight unbiased and objective statements such as: "*GRACE data did not provide any indication that the performance of 90-day feeding studies (following OECD or EFSA guidelines and current practice) with whole food/feed would provide additional information on the safety of maize MON810 when compared to the compositional comparison of the GM line and its closest conventional comparator in terms of an initial comparative safety assessment.*"(1). Prima facie this statement (directed to risk managers) appears to be clear: there is no sense in conducting 90-day feeding studies with whole food/feed. But the specialty lies in the detail: The data <u>they have got</u> do not indicate any justification for performing such studies. And in this case the conclusion allows two diametrically different interpretations:

a) The tests have been performed *lege artis* and all has worked as expected, there is good evidence that there is no sense in conducting 90-day feeding studies.

b) Because of shortcomings during the execution of the animal experimentation (e.g. a potential high background/laboratory-induced variation) the GRACE consortium could not find any indication in support for performing such feeding studies which may otherwise have shown some effects if laboratory-induced variation would have been kept low.

There are indications that the latter interpretation may have some credibility considering the shortcomings in the animal experimentation (e.g. circadian effects, need for conventional varieties to generate historical controls etc.)

The response given by GRACE to our initial comment is even more carefully formulated by saying "*Specific and detailed discussions are and will be provided within the scientific publications. GRACE also underlines that currently a detailed overview of research data on the added value of a range of methods is not available…. Especially the lack of a positive control for whole food/feed is challenging. Therefore, GRACE urges a broader scientific discussion based on accessible data.*" Although this should have already been clearly mentioned in the final "Conclusions and Recommendations" we appreciate the proposed broader scientific discussion based on accessible data. That sounds promising because at present the requirement for a systematic identification of sources of uncertainty has been widely ignored by the GRACE consortium (for additional information please see also our reply to comment 3).

10. Reply to the GRACE response to our comment 14 concerning the unavailability of all feeding trial data for a broad discussion and the role of the CADIMA database.

It is highly appreciated that the CADIMA database has been introduced as a platform supporting GMO risk assessment. CADIMA (http://www.cadima.info/index.php) is said to "provide relevant background information in relation to the risk assessment process and access to raw data generated by associated research activities" such as GRACE.

With respect to the GRACE animal feeding trials currently only the data of the two trials A and B - as confirmed by the GRACE consortium - are provided by CADIMA (database last accessed: June 3rd, 2016). Data of the other three feeding studies (C, D, and E) are still missing. Therefore, the CADIMA database in its present condition does not fulfill its intended function as the intended platform (i.e. as basis for a public and open discussion) and is in our opinion not an adequate source of an ad hoc informed decision making. This can only be envisioned if all relevant data retrieved in the course of the GRACE project have been published and stored in CADIMA.

11. Reply to the GRACE response to our comment 15.1 concerning shortcomings in the overall study approach of trial A and B and their inadequate communication in the final "Conclusions and Recommendations".

The GRACE consortium is considering a completely different class of limitations in their response as what was intended by our input. We do not refer to "intrinsic" limitations of 90-day feeding studies with whole GM food and feed but to limitations of the overall approach and the lack of a clear communication of these inadequacies in the final "Conclusions and Recommendations".

It is noticeable that - concerning 90-day feeding studies - the term "limitation" appears only once in the "Conclusions and Recommendations" (p.31; (1)); and this in the context of "intrinsic" limitations which are not even elaborated in the relevant part of the manuscript (Part I; pages 20 – 31; (1)). An explicit listing of "strengths and limitations" of 90-day feeding trials in rodents as announced in the "Introduction to GRACE" (p. 6; (1)) plus a discussion of the limitations of the overall approach as indicated in our comment would have been essential for an informed decision-making - but are missing from the final "Conclusions and Recommendations" (1).

For clarification we repeat the list of general limitations of their approaches and provide additional information:

1. The Grace consortium checked only a single transgenic event (Cry1Ab; MON810) (1). This is not an "intrinsic" limitation of a 90-day feeding study with whole GM food and feed but a deliberate decision of the consortium with certain implications:
   a) The data and conclusions generated thereof are only valid for the given test setting. No other modes of action concerning transgenic events have been experimentally checked.
   b) The justification for a broad generalization of the conclusions drawn from the tested Cry1Ab-MON810 - 90-day feeding study in rats for other transgenic elements, events, modes of actions, plant lines etc. has not been demonstrated in the course of the GRACE project. The general applicability of conclusions drawn for single events to substantially modified GM plants still remains to be elusive (5, 6, 42, 43). In this respect the envisioned feeding studies with transgenic potatoes (p. 8; (1)) would have brought extra information and would have added substantially to the overall credibility of the final "Conclusions and Recommendations".
   c) This limitation has to be clearly communicated (2).
2. The GRACE consortium did not perform studies with substantially altered transgenic plants (e.g. Second Generation GMOs) in 90-day whole food/feed studies in rodents. Therefore, GRACE cannot provide empirical evidence that their conclusions apply also to these substantially genetically modified products. Such limitation has to be clearly communicated in the final "Conclusions and Recommendations" (2)
3. The GRACE consortium appears to have not analyzed the data obtained from trial A and B for **trends** and **tendencies** in the retrieved results. However, determining and discussing trends in the obtained data packages is an integral element of the toxicological risk assessment of whole food and feed animal testing. EFSA is of the opinion that the involvement of special experts can *"…help with the detection of* ***trends****…"*, that cluster analysis may be applied to "… *visualize* ***central tendency***" of data, and that the first step in data analysis should include a visual inspection of the data *"…for* ***trends*** *and patterns…"* (5). To evaluate whether a relationship with treatment is causal or not the determination of "*…dose-related* ***trends*** *or relationships…*" represents a valuable element in toxicological risk assessments (5). EFSA subsequently reiterated that the identification of *"…possible* ***trends..***." is an element to be considered for the interpretation of the results of animal studies (7). This limitation – the omission of a trend analysis - should to be clearly communicated in the final "Conclusions and Recommendations" (2).

We are concerned by the omission of vital information necessary for informed decision-making in the final "Conclusions and Recommendations" (2). This is a communication problem. GRACE does not communicate uncertainties, limitations and shortcomings of their overall approach to an audience (i.e. primarily risk managers) vitally relying on this kind of information.

12. Reply to the GRACE response to our comment 15.2 concerning the lack of information on GMOs with stacked events or with complex alterations of metabolic pathways.

In its reply GRACE insinuates that we assume *"… that a (90-day) feeding trial has a definite added value in the cases listed above*" and that this would imply *"… that these GM plants will show more pronounced effects*" and that *"…. Regarding the intrinsic limitations of 90-day feeding trials with whole food/feed, the Comment underlines the recommendations made by GRACE.*"

This statement indicates that GRACE is not of the opinion that substantially modified GM plants deserve a special attention concerning their toxicological assessment by 90-day feeding studies. We would like to draw the attention of the GRACE consortium to the position of the following experts who apparently expect more pronounced effects and indeed appear to see a definite added value of 90-day feeding trials for substantially modified GM plants:

Kuiper et al. anticipate that *"…Safety testing will have to be adjusted for the "**second generation**" of food plants, which are modified to improve food-quality traits—e g, to raise the nutritional value of the proteins, to increase concentrations of oils low in saturated fats or of novel carbohydrates, or to fortify the foods with micronutrients or antioxidants. These must undergo extensive toxicological and nutritional assessment with a combination of in-vitro and **in-vivo** techniques as required for novel foods in general." (42)*

Hans Christer Andersson, Detlef Bartsch, Hans-Joerg Buhk, Howard Davies, Marc De Loose, Michael Gasson, Niels Hendriksen, Colin Hill, Sirpa Kärenlampi, Ilona Kryspin-Sørensen, Harry Kuiper, Marco Nuti, Fergal O'Gara, Pere Puigdomenech, George Sakellaris, Joachim Schiemann, Willem Seinen, Angela Sessitsch, Jeremy Sweet, Jan Dirk van Elsas and Jean-Michel Wal are of the opinion that *"..If the composition of the GM plant is **modified substantially**, or if there are any indications for the potential occurrence of unintended effects, based on the preceding molecular, compositional or phenotypic analysis, not only new constituents, but also the whole GM food/feed should be tested. In such a case, the testing programme should include at **least a 90-day toxicity*** study in rodents.*"(43)*

Gerrit Alink, Sue Barlow, Andrew Cockburn, Gerhard Flachowsky, Ib Knudsen, Harry Kuiper, Dominique Parent Massin, Gerard Pascal, Ad Peijnenburg, Richard Phipps, Annette Pötting, Morten Poulsen, Willem Seinen, Horst Spielmann, Henk van Loveren, Jean-Michel Wal, Anthony Williams state that *"…If the composition of the new GM plant or derived food or feed is **modified substantially** or if there are any indications of untoward effects, **animal studies** should also be conducted on the relevant food or feed matrix."* (5) The same authors confirm that *"…The use of **90-days studies in rodents** should be considered for the detection of possible unintended effects in food and feed derived from GM plants which have been **more extensively modified** in order to cope with environmental stress conditions like drought or high salt conditions, or GM plants with quality or output traits with the purpose to improve human or animal nutrition and/or health."*(5)

Hans Christer Andersson, Salvatore Arpaia, Detlef Bartsch, Josep Casacuberta, Howard Davies, Patrick du Jardin, Gerhard Flachowsky, Lieve Herman, Huw Jones, Sirpa Kärenlampi, Jozsef Kiss, Gijs Kleter, Harry Kuiper, Antoine Messéan, Kaare Magne Nielsen, Joe Perry, Annette Pöting, Jeremy Sweet, Christoph Tebbe, Atte Johannes von Wright, and Jean-Michel Wal add that *"…If the composition of the food and/or feed derived from GM plant is **substantially modified**, or if there are any indications for the potential occurrence of unintended effects based on the preceding molecular, compositional or phenotypic analyses, not only new constituents but also the **whole food and feed** derived from the GM plant should be tested. In such case the testing program should include a **90-day toxicity study** in rodents."(6)*

This is a non-exhaustive list of renowned experts in the field of GMO risk assessment covering the scientific consensus on this issue over the past decade. It is notable that members of the GRACE consortium including the coordinator of the GRACE project are on this list of authors who apparently indeed see a definite added value of 90-day feeding trials for substantially modified GM plants (6, 43).

In this context we would like to recall that the detection of unintended effects is one of the primary reasons to perform a 90-day whole food/feed study in rodents (5, 6, 42-45). These unintended effects may also comprise "unexpected" effects (5, 10, 46). Referring to the suggestion of the GRACE consortium that under the mentioned conditions *"…a targeted approach should be considered using an appropriate study design…"* we would like to indicate that it is extremely challenging to develop a targeted approach including a hypothesis-driven study design for effects which are – per definitionem – unexpected.

It is obvious that in the framework of GRACE not all of the indicated genetic variations of GM plants could have been tested nor was this requested from GRACE in our comment. However, the restricted variety of the test items is a limitation of the approach (not an "intrinsic" limitation of 90-day feeding trials in rats) which the GRACE consortium should communicate clearly in their final "Conclusions and Recommendations" if they want to provide an unbiased guide for an informed decision-making (2).

Consequently we cannot follow the argumentation of the GRACE consortium that our comment as presented in section 15.2 should underline their recommendations, as we simply indicated significant shortcomings and omissions in the **presentation** of the final "Conclusions and Recommendations".

13. Reply to the GRACE response to our comment 15.3 concerning the quality requirements for animal testing facilities to obtain meaningful results from 90-day feeding studies.

The GRACE consortium concedes that *"...experience in trials performed with food/feed"* was lacking before the initiation of the GRACE project. The comment in section 15.3 is exclusively targeting this situation at that specific location concerning whole food/feed studies with rats. The necessity to include reference varieties into the test design substantiates the lack of existing expertise in the field of whole food/feed studies with rats. The inclusion of reference varieties is discouraged and only acceptable according to EFSA if the executing laboratory cannot provide historical data. EFSA is of the opinion that "…in the case of GM food, the inclusion in the experimental design of reference groups, fed with a diet containing commercially available food/feed similar to the test food/feed … is in general not recommended…" and that "…historical background data on variations in endpoint values should primarily be obtained from databases available in the actual testing facility…" (7).

It is remarkable that the GRACE consortium initiated a project with a partner laboratory - responsible for the proper execution of the core element and the cornerstone of the GRACE project - which actually had no experience in performing the core task (i.e. the execution of the 90-day whole food and feed studies with maize in rats: *"…the institution conducting the feeding trials, had not performed such studies with maize in the past, and therefore, appropriate historical data with which one could compare the results obtained in the present study were lacking."* (11)).

14. Reply to the GRACE response to our comment 15.4 concerning the non-detectability of the Cry1Ab protein in some of the transgene containing diets.

We agree with the rebuttal of the GRACE consortium. Some of the provided information appears to have been mixed up.

GRACE is indicating that *"…in contrast, the cry1Ab gene could have not been amplified by PCR for some diets*". We are in line with GRACE that this is discomforting and that it can be an indication for mutations/deletions/truncations in the primer binding regions of cry1Ab-specific PCRs (leading in the worst case to aberrant Cry protein variants) or for inadequate template DNA purification. In our opinion both situations could have easily been solved by either re-designing appropriate in house PCR primers or by applying an efficient DNA extraction procedure to clarify the knowledge gap. In this context it has to be noted that a PCR procedure for detection and quantification of the MON810 event has been validated by JRC and should be available in all national GMO reference laboratories (http://gmo-crl.jrc.ec.europa.eu/StatusOfDossiers.aspx).

15. Reply to the GRACE response to our comment 15.5 concerning the presence of the Cry1Ab in conventional control varieties.

The GRACE consortium is of the opinion that "…*The statement by the Austrian colleagues is not precise.*" Our comment was obviously misunderstood because of an indistinct wording. Our concerns were referring exclusively to the allegedly "GM-free" non-transgenic conventional control diets 1 of trial A and B which have actually been GM-diets:

The conventional control diet 1 consisting of the non-GM variety PR32T83 used in trial B contained 2.6% of genetically modified DNA (Table 2; Zeljenkova et al., 2014) (11). According to Regulation (EC) 1830/2003 this diet has to be labelled as genetically modified ("this product contains genetically modified organisms") (47). The conventional control diet 1 consisting of the non-GM variety PR33W82 used in Trial A displayed a GMO content of 1.2% (Table 2; Zeljenkova et al., 2014, (11)) and, thus, surpassed the legal threshold for GM-labelling, too (47, 48). For both conventional non-transgenic maize control varieties levels of contamination with the Cry1Ab protein have been quantified: Both conventional non-transgenic control diets contained approx. 10% of the Cry1Ab toxin protein of the 11% GMO verum group (e.g. conventional 1, trial B: 0.18 ng/mg vs 11% GMO: 2.01 ng/mg; Table 2; Zeljenkova et al., 2014, (11)). These GM contaminated conventional control diets may therefore be considered as a third GM-diet in the trials, which in fact have been performed with three GM-containing diets (i.e. approx. 1%, 11% and 33% GMO content).

No quantitative data for the contamination of the non-GM near-isogenic comparator containing diet with the Cry1Ab toxin were presented by GRACE although genetic elements of the MON810 event have been detected in this control diet (11). Therefore, we did not assess the potential effect of these contaminations on the outcome of trials A and B. Notably, the GRACE consortium is referring to a difference of more than 2 orders of magnitude in the GM content between verum and the contaminated near isogenic comparator containing diets. We would like to indicate that in molecular biology the number of distinct molecules which are transferred into a biological system is of importance. The amount of 1 ng MON810-derived pure Cry1Ab protein contains approx. 6.6×10^9 Cry1Ab molecules (Cry1Ab: 91 kD (49); number of particles in 1 ng protein: $6.022 \times 10^{14}/91000=6.6 \times$

10^9). Maybe that in the classical toxicological mode of thinking a reduction of the dose to 1/100 may lead to coming below a potential effect. But there are examples where due to (signal) amplification (for instance with immunogenic effects and specifically presumed in this case; for details see comment 2 and references therein) even very small doses are able to play a not insignificant role. In this sense, a reduction in the number of molecules from 10^9 Cry1Ab protein particles to 10^7 (= 2 orders of magnitude) would not really be satisfactory in terms of a substantial elimination of the active principle from the test system.

The observation of GM-contamination of conventional control varieties containing diets is of significance because the GRACE consortium is frequently referring to the results of these "non"-transgenic diets (= surrogates for historical controls) when they argue away statistically significant differences between verum and near-isogenic non-GM comparator treatment groups (e.g.: [Haematology parameters] *"…were within or close to the ranges of the groups fed the two conventional maize varieties.*"; "*The ALT activity in the 11 % GMO group is close to the range of the groups fed conventional maize varieties.*"; [GLU, CHOL and TRG levels]: *"…and that the measured values were within or close to the ranges of the groups fed the conventional maize varieties…"*, " *…Ca, K and P values were within or close to the ranges of the groups fed the conventional maize varieties*" etc…) (11). Consequently, it is scientifically questionable to take into account these GM-contaminated "conventional" diets for establishing the range of "naturally" occurring biological variation or as "historical control" for laboratory-associated variation during the course of 90-day feeding trials.

16. Reply to the GRACE response to our comment 15.6 concerning stakeholder engagement and the absence of any hints on discordance in the final "Conclusions and Recommendations."

In our comment we have been addressing shortcomings in the mode of communication and on how the GRACE consortium is delivering its message. The final "Conclusions and Recommendations" are primarily targeted on and shaped for policy- and decision-makers. These policy- and decision-makers might not all be longstanding experts in toxicology and animal experimentation, nor are they necessarily aware of all pitfalls, limitations and uncertainties associated with the issues the GRACE consortium is dealing with. We would like to underline that the term "uncertainty" comprises all types of limitations in the knowledge available to assessors at the time an assessment is conducted and within the time and resources available for the assessment (2). And it is the key task of the assessors to inform decision-makers about scientific uncertainty when providing their advice or vice versa how probable the drawn conclusions are considering their limitations (2). The final "Conclusions and Recommendations" dealing with the 90-day feeding studies in rats fail to comply with these requirements. The GRACE consortium must not expect decision makers - who rely on an unbiased presentation of the topic in exactly these final "Conclusions and Recommendations" - to scan several gigabytes of information spread over various webpages, databases, stakeholder reports, open letters and scientific publications, where all these points of criticism may or may not have been addressed, and get an authentic opinion out of those. The GRACE "Conclusions and Recommendations" may indeed be based on extensive internal discussions. However, this circumstance – and that is the central point of criticism - is not reflected in this published document.

It is obvious that the final "Conclusions and Recommendations" are not the forum for a detailed reiteration of stakeholder concerns. However, in our opinion it is the duty of the GRACE consortium to clearly address the limitations and uncertainties in their line of argumentation in the booklet. This is a basic requirement for assessors who are providing scientific advice for decision-makers (2).

17. Reply to the GRACE response to our comment 15.7 concerning the mandatory requirement of 90-day feeding studies if concerns are raised during comparative analyses of a GM plant.

The confirmation of our input by the GRACE consortium is acknowledged.

18. Reply to the GRACE response to our comments 15.8 and 15.9 concerning trends and the role of conventional control varieties in this context

The GRACE consortium is of the opinion that our line of argumentation is inconsistent when we suggest using these additional control data to detect trends in the data packages "…. *while initially suggested not to include such data.*"

For clarification we would like to emphasize again that in toxicological risk assessments – also with GMOs - predominantly the dose groups have to be compared with the control which is kept under the same conditions but without the interesting/test substance/s. But if – for whatever reasons – some other groups are employed, we are of the opinion that it is justified to take a look at these data and to check them also for potentially emerging trends (e.g. ranging over all other groups as against the verum groups or vice versa and, thus, distinguishing the test groups from all other groups). The GRACE consortium itself takes advantage of conventional control data to dismiss – inter alia – statistically significant differences between test groups and the non-GM near isogenic control as not pathologically relevant so these data should be also eligible for the evaluation of (also statistically non-significant) tendencies in the relevant data packages. In our opinion this approach would provide valuable additional insights and should be systematically considered for the sake of gaining as much information as possible from the experiment. However, we want to reiterate that we do not request the inclusion of conventional controls in the test design and that we are completely in line with EFSA's statement that "*…in the case of GM food, the inclusion in the experimental design of **reference groups**, fed with a diet containing commercially available food/feed similar to the test food/feed … is in general **not recommended…***" (7).

It is obvious that the GRACE consortium puts a certain focus on the relevance of historical control data and the inclusion of conventional varieties in their test design. The importance of conventional varieties for the GRACE consortium is reflected in the "Conclusions and Recommendations" where a total of three paragraphs (p. 23 – 24; (1)) are reserved for discussing the advantages of conventional varieties whereas ("intrinsic") limitations of 90-day feeding trials, uncertainties, and trends in the obtained data are not at all explicitly elaborated.

In Zeljenkova et al., 2014, GRACE is referring at five occasions to conventional control varieties to confirm a non-biological relevance of the observed aberrant results (11). Adding the 5 occasions where it is referred to historical control data as supplied by the producer of the rat strain (Harlan) this type of argumentation is used more than 100% more often than the third most frequent

argument ("no dose dependency": 4 times). The information was retrieved from a quantitative analysis of the lines of reasoning to argue away a biological relevance of significant differences between GM and non-GM fed groups as applied in the discussion of Zeljenkova et al., 2014 (11).

Werner Brüller
Walter Stepanek
Markus Wögerbauer

Department for Integrative Risk Assessment, Data and Statistics
Austrian Agency for Health and Food Safety
Spargelfeldstrasse 191
1220 Vienna, Austria

References

1. **GRACE Consortium.** 2015. Conclusions and recommendations on animal feeding trials and alternative approaches and on the use of systematic reviews and evidence maps for GMO impact assessment. http://www.grace-fp7.eu/de/content/grace-published-its-final-conclusions-and-recommendations.
2. **EFSA Scientific Committee.** 2016. Guidance on Uncertainty in EFSA Scientific Assessment. Draft. EFSA Journal.
3. **European Commission.** 2001. Directive 2001/18/EC of the European Parliament and of the Council of 12 March 2001 on the deliberate release into the environment of genetically modified organisms and repealing Council Directive 90/220/EEC, p. 1-38, L 106/1.
4. **European Commission.** 2003. Regulation (EC) No 1829/2003 of the European Parliament and of the Council of 22 September 2003 on genetically modified food and feed of 22 September 2003 on genetically modified food and feed, p. 1-23, L 268/1.
5. **EFSA.** 2008. Safety and nutritional assessment of GM plants and derived food and feed: the role of animal feeding trials. Food Chem Toxicol **46 Suppl 1:**S2-70.
6. **EFSA.** 2011. Guidance for risk assessment of food and feed from genetically modified plants. EFSA Panel on Genetically Modified Organisms (GMO) The EFSA Journal **9:**2150.
7. **EFSA.** 2011. Guidance on conducting repeated-dose 90-day oral toxicity study in rodents on whole food/feed. The EFSA Journal **9:**2438 - 2459.
8. **Pielaat A, Barker GC, Hendriksen P, Hollman P, Peijnenburg A, Kuile BH.** 2013. A foresight study on emerging technologies: State of the art of omics technologies and potential applications. REPORT 1: Review on the state of art of omics technologies in risk assessment related to food and feed safety. EFSA supporting publication., p. 1-126, vol. EN-495.
9. **Pauwels K, De Keersmaecker SCJ, De Schrijver A, du Jardin P, Roosens NHC, Herman P.** 2015. Next-generation sequencing as a tool for the molecular characterisation and risk assessment of genetically modified plants: Added value or not? Trends Food Sci Technol **45:**319-326.
10. **Devos Y, Naegeli H, Perry JN, Waigmann E.** 2016. 90-day rodent feeding studies on whole GM food/feed: Is the mandatory EU requirement for 90-day rodent feeding studies on whole GM food/feed fit for purpose and consistent with animal welfare ethics? EMBO Rep **17:**942-945.
11. **Zeljenkova D, Ambrusova K, Bartusova M, Kebis A, Kovriznych J, Krivosikova Z, Kuricova M, Liskova A, Rollerova E, Spustova V, Szabova E, Tulinska J, Wimmerova S, Levkut M, Revajova V, Sevcikova Z, Schmidt K, Schmidtke J, La Paz JL, Corujo M, Pla M, Kleter GA, Kok EJ, Sharbati J, Hanisch C, Einspanier R, Adel-Patient K, Wal JM, Spok A, Poting A, Kohl C, Wilhelm R, Schiemann J, Steinberg P.** 2014. Ninety-day oral toxicity studies on two genetically modified maize MON810 varieties in Wistar Han RCC rats (EU 7th Framework Programme project GRACE). Arch Toxicol **88:**2289-2314.
12. **OECD.** 1998. Guideline for the testing of chemicals - Repeated Dose 90-day Oral Toxicity Study in Rodents. OECD.
13. **European Commission.** 2004. Directive 2004/10/EC of the European Parliament and of the Council of 11 February 2004 on the harmonisation of laws, regulations and administrative provisions relating the application of the principles of good laboratory practice and the verification of their applications for tests on chemical substances. Off. J. Eur. Communities **L 50:**44–59.
14. **European Commission.** 2013. Commission Implementing Regulation (EU) No 503/2013 of 3 April 2013 on applications for authorisation of genetically modified food and feed in accordance with Regulation (EC) No 1829/2003 of the European Parliament and of the Council and amending Commission Regulations (EC) No 641/2004 and (EC) No 1981/2006 Text with EEA relevance. Available from http://eur-lex.europa.eu/legal-content/EN/TXT/?uri=CELEX:32013R0503.

15. **EC.** 2003. Regulation (EC) No 1830/2003 of the European Parliament and of the Council of 22 September 2003 concerning the traceability and labelling of genetically modified organisms and the traceability of food and feed products produced from genetically modified organisms and amending Directive 2001/18/EC, p. 24-28, Official Journal of the European Communities, vol. L 268.
16. **Haas J, Manro J, Shannon H, Anderson W, Brozinick J, Chakravartty A, Chambers M, Du J, Eastwood B, Heuer J, Iturria S, Iversen P, Johnson D, Johnson K, O'Neill M, Qian HR, Sindelar D, Svensson K.** 2004. In Vivo Assay Guidelines. *In* Sittampalam GS, Coussens NP, Nelson H, Arkin M, Auld D, Austin C, Bejcek B, Glicksman M, Inglese J, Iversen PW, Li Z, McGee J, McManus O, Minor L, Napper A, Peltier JM, Riss T, Trask OJ, Jr., Weidner J (ed.), Assay Guidance Manual, Bethesda (MD).
17. **GRACE Consortium.** 2015. GRACE: One-year oral toxicity study on a genetically modified maize MON810 variety in Wistar Han RCC rats. (EU 7th Framework Programme project GRACE). Supporting documentation (53 pages). October 2015. Steinberg_supporting doc.pdf. http://backup.grace.jki.bund.de/.
18. **GRACE Consortium.** 2015. Non-Disclosure Agreement: (page 4; section 3.12.). Distributed by email on 16.9.2015
19. **Clayton JA, Collins FS.** 2014. Policy: NIH to balance sex in cell and animal studies. Nature **509:**282-283.
20. **American Diabetes Association.** 2008. Diagnosis and Classification of Diabetes Mellitus. Diabetes Care **31:**562 - 567.
21. **Shrayyef MZ, Gerich JE.** 2010. Normal Glucose Homeostasis, 2nd ed.
22. **Woerle HJ, Meyer C, Popa EM, Cryer PE, Gerich JE.** 2003. Renal compensation for impaired hepatic glucose release during hypoglycemia in type 2 diabetes: further evidence for hepatorenal reciprocity. Diabetes **52:**1386-1392.
23. **Stumvoll M.** 1998. Glucose production by the human kidney - its importance has been underestimated. Nephrol Dial Transplant **13:**2996-2999.
24. **McMullin MF.** 2010. Diagnosis and treatment of erythrocytosis, p. 55-58, European Haematology, vol. 4.
25. **Hoffbrand V, Moss PAH.** 2016. Hoffbrand's essential haematology, 7th Edition. Wiley.
26. **Finamore A, Roselli M, Britti S, Monastra G, Ambra R, Turrini A, Mengheri E.** 2008. Intestinal and peripheral immune response to MON810 maize ingestion in weaning and old mice. J Agric Food Chem **56:**11533-11539.
27. **Sompayrac LM.** 2015. How the immune system works, 5th Edition. Wiley.
28. **Sagstad A, Sanden M, Haugland O, Hansen AC, Olsvik PA, Hemre GI.** 2007. Evaluation of stress- and immune-response biomarkers in Atlantic salmon, Salmo salar L., fed different levels of genetically modified maize (Bt maize), compared with its near-isogenic parental line and a commercial suprex maize. J Fish Dis **30:**201-212.
29. **Walsh MC, Buzoianu SG, Gardiner GE, Rea MC, Gelencser E, Janosi A, Epstein MM, Ross RP, Lawlor PG.** 2011. Fate of transgenic DNA from orally administered Bt MON810 maize and effects on immune response and growth in pigs. PLoS One **6:**e27177.
30. **Walsh MC, Buzoianu SG, Rea MC, O'Donovan O, Gelencser E, Ujhelyi G, Ross RP, Gardiner GE, Lawlor PG.** 2012. Effects of feeding Bt MON810 maize to pigs for 110 days on peripheral immune response and digestive fate of the cry1Ab gene and truncated Bt toxin. PLoS One **7:**e36141.
31. **Andreassen M, Rocca E, Bøhn T, Wikmark O-G, van den Berg J, Løvik M, Traavik T, Nygaard UC.** 2015. Humoral and cellular immune responses in mice after airway administration of Bacillus thuringiensis Cry1Ab and MON810 cry1Ab-transgenic maize. Food Agric Immunol **26:**521-537.
32. **EFSA.** 2011. Guidance of the EFSA Scientific Committee on conducting repeated-dose 90-day oral toxicity study in rodents on whole food/feed. The EFSA Journal **9(12):2438:**1-21.

33. **OECD.** 2008. Guideline for the testing of chemicals - Repeated Dose 28-Day Oral Toxicity Study in Rodents OECD.
34. **Guzman-de la Garza FJ, Ibarra-Hernandez JM, Cordero-Perez P, Villegas-Quintero P, Villarreal-Ovalle CI, Torres-Gonzalez L, Oliva-Sosa NE, Alarcon-Galvan G, Fernandez-Garza NE, Munoz-Espinosa LE, Camara-Lemarroy CR, Carrillo-Arriaga JG.** 2013. Temporal relationship of serum markers and tissue damage during acute intestinal ischemia/reperfusion. Clinics (Sao Paulo) **68:**1034-1038.
35. **EFSA.** 2014. Explanatory statement for the applicability of the Guidance of the EFSA Scientific Committee on conducting repeated-dose 90-day oral toxicity study in rodents on whole food/feed for GMO risk assessment. The EFSA Journal **12(10):3871:**1-25.
36. **Falcone FH, Haas H, Gibbs BF.** 2000. The human basophil: a new appreciation of its role in immune responses. Blood **96:**4028-4038.
37. **EFSA.** 2012. Guidance of the ANS Panel for submission for food additive evaluations. The EFSA Journal **10(7):2760:**1-60.
38. **Smets G, Adduci G, Spök A.** 2015. Assessing Feeding Studies and Alternative Approaches for GMO Risk Assessment Stakeholder Consultation on the results of the GRACE feeding studies as well as of the in-vitro, in-silico, and analytical studies and on general conclusions and recommendations. Report 30 November 2015.
39. **Ellis PD.** 2010. The essential guide to effect sizes: statistical power, meta-analysis, and the interpretation of research results. University Press, Cambridge, Cambridge.
40. **Adduci G, Spök A.** 2015. GRACE Stakeholder Consultation on Animal Feeding Studies and In Vitro Studies in GMO Risk Assessment. Draft Results of the 90-day Animal Feeding Studies and Progress of the In-Vitro, In-Silico and Analytical Studies with GM Maize. Report June, 2015.
41. **van Dijk JP, de Mello CS, Voorhuijzen MM, Hutten RC, Arisi AC, Jansen JJ, Buydens LM, van der Voet H, Kok EJ.** 2014. Safety assessment of plant varieties using transcriptomics profiling and a one-class classifier. Regul Toxicol Pharmacol **70:**297-303.
42. **Kuiper HA, Noteborn HPJM, Peijnenburg AACM.** 1999. Adequacy of methods for testing the safety of genetically modified foods. The Lancet **354:**1315-1316.
43. **EFSA.** 2006. Guidance document of the Scientific Panel on Genetically Modified Organisms for the risk assessment of genetically modified plants and derived food and feed. The EFSA Journal **99:**1-100.
44. **European Commission.** 2003. The risk assessment of genetically modified plants and derived food and feed. Prepared for the EU Scientific Steering Committee by the Joint Working Group on Novel Foods and GMOs. EC Guidance Document, 6-7 March 2003. Commission of the European Communities, Brussels.
45. **GM Science Review Panel.** 2003. GM Science Review. First Report 6704/0. Department of Trade and Industry, London, pp. 92–96. <http://www.gmsciencedebate.org.uk/report/pdf/gmsci-report1-full.pdf>.
46. **Cellini F, Chesson A, Colquhoun I, Constable A, Davies HV, Engel KH, Gatehouse AMR, Kärenlampi S, Kok EJ, Leguay JJ, Lehesranta S, Noteborn HPJM, Pedersen J, Smith M.** 2004. Unintended effects and their detection in genetically modified crops. Food Chem Toxicol **42:**1089-1125.
47. **European Commission.** 2003. Regulation (EC) 1830/2003 of the European Parliament and of the Council of 22 September 2003 concerning the traceability and labelling of genetically modified organisms and the traceability of food and feed products produced from genetically modified organisms and amending Directive 2001/18/EC. Off. J. Eur. Communities **L268:** 24–28.
48. **National Austrian GMO Reference Laboratory.** 2016. Personal communication on March 16th, 2016.
49. **CERA.** 2016. GM Crop Database: MON810. http://www.cera-gmc.org/GmCropDatabaseEvent/MON810/pdf. Last accessed April 26th, 2016.
